# Clean Teeth

**Published:** 1875-04

**Authors:** 


### CLEAN TEETH.

The moral which we have endeavored to point in our little articles on the Teeth, is this: That strict cleanliness secures perfect teeth; that uncleanness just as surely produces unsound teeth; and that nothing less than daily care and thoughtful attention can be signified with the title of cleanliness. When the writer of this paragraph was a child, his mother provided him with brushes and dentifrices, and urged him to use them faithfully each day. She never *insisted* upon it, however, except on Sunday, and then she stood by and saw it done. So our teeth got a good scrubbing once a week, if no more, and this helped a good deal. If the good woman had devoted a couple of minutes each evening to the superintendence of this important work, and had followed it up while we were under her care, we need never have lost any one of the half-dozen teeth whose extraction we now lament. If, in our boyhood, we could have been supplied with plenty of Dr. Lyon's beautiful Tooth-Tablets, and had been forced to use them with a stiff brush each night on retiring, we never should have lost a tooth from decay. What is true of ourselves is equally true of nine-tenths of the human race.

The fact is, the so-called "civilized" world is worse than barbarians in this respect. Chinamen of the better class take scrupulous care of their teeth. The teeth of the Japanese are of a superior character,—much finer than our own. All but the very low and ignorant among them are cleanly; and to keep their teeth clean is a part of their religious duty. This duty they perform each morning and each night before going to prayers. They first clean the tongue and then the teeth, and then proceed to offer up prayer. With them, far more than with many Christians, cleanliness is next to Godliness. In fact, with them cleanliness *is* Godliness, for it is a part of their morning and evening devotions. Can we not so far impress our readers with the importance of care in the direction of the teeth that they will consider what we have to say from month to month, and strive to act upon our suggestions?

---

Lung-Power.—It has been ascertained that the extreme height of a column of water, supported by the muscular act of expiration transmitted by the lips, is about six feet. Experiments with a view to discover what is the actual pressure corresponding to the full production of a note on each of the principal wind-instruments show, that, with the majority of wind-instruments, the pressure required by the high notes is considerably greater than that required for the low notes, each particular description of instrument being found to have a presure-ratio peculiarly its own. The clarionet, however, is mentioned as an exception to this rule.

---

Young women should reflect that it is a thousand times better to marry a *man* without wealth than to marry *wealth* without a man. The wealth can be acquired afterward by honest industry and economy; but, no power can create a man out of the mere human thing which has been married for his wealth.

